# Movements and spatial usage of harbour seals in the Elbe estuary in Germany

**DOI:** 10.1038/s41598-023-33594-1

**Published:** 2023-04-24

**Authors:** Abbo van Neer, Dominik Nachtsheim, Ursula Siebert, Thomas Taupp

**Affiliations:** 1 Institute for Terrestrial and Aquatic Wildlife Research, University of Veterinary Medicine Hannover, Foundation, Werftstraße 6, 25761 Büsum, Germany; 2 Department of Animal Ecology, Federal Institute of Hydrology (BfG), Am Mainzer Tor 1, 56068 Koblenz, Germany

**Keywords:** Ecology, Behavioural ecology, Marine biology

## Abstract

Harbour seals are top predators in the North Sea and regarded as sentinels for ecosystem health. A few hundred also occur in adjacent estuaries, such as the Elbe estuary, Germany. However, only little is known about how these animals use this dynamic tidally influenced habitat, which has been under high anthropogenic pressure for decades. In this context, nine harbour seals (*Phoca vitulina*) from the Elbe estuary were equipped with biotelemetry devices to track their movements over multiple months. Harbour seal movements were characterised by short trips (trip length outside pupping season for females: 9.0 ± 1.12 km, males: 7.0 ± 1.24 km) as well as small home ranges (median 50% home range for females: 16.3 km^2^, males: 36.1 km^2^) compared to harbour seals from marine regions. Within the estuary, the animals utilised the fairway, river branches and tributaries. During the pupping season in June and July, four seals showed strongly reduced trip lengths and durations, increased daily haul out durations as well as smaller home ranges. Even though a continuous exchange with harbour seals from the Wadden Sea likely occurs, most individuals in this study spent the entire deployment duration inside the estuary. This indicates that the Elbe estuary provides a suitable habitat for harbour seals, despite extensive anthropogenic usage, calling for further studies on the consequences of living in such an industrialised habitat.

## Introduction

Harbour seals (*Phoca vitulina*) are one of the key species in the North Sea and regarded as one of the top predators of the ecosystem besides the grey seal (*Halichoerus grypus*) and the harbour porpoise (*Phocoena phocoena*)^1–3^. Within the North Sea, harbour seals occur in a diverse set of habitats from rocky shores around the United Kingdom (UK) to the tidal mudflats of the Wadden Sea^4,5^. The harbour seal population in the Wadden Sea, occurring between Den Helder in the Netherlands and Esbjerg in Denmark (for details, see map in Figure A. 1 in the Supplementary materials), is counted annually by aerial surveys, with the latest moult survey amounting to 23,652 counted individuals in August 2022^6^. The species shows diverging trends in abundance throughout its European range, with some areas stable or increasing, and others suffering serious declines, e.g. the Orkney Isles and East Scotland^1,4^. Numbers in the Wadden Sea have in contrast stabilised at a high level over the last years, potentially indicating carrying capacity induced growth limitation^1,7^. With 13,304 individuals counted in 2022 (56% of the Wadden Sea population), the German part of the Wadden Sea is considered a stronghold for the species in the region^6^.

The characteristic physical feature of the Wadden Sea is the semi-diurnal lunar tide, which regularly exposes sandbanks in the tidal mudflats^8^. During these times, seals use the available substrate and haul out in order to rest. Apart from resting, these tidally dependent haul outs are also utilised more frequently in June and July for giving birth to the pups as well as during August for moulting^5,9–11^. The haul out sites used by harbour seals are usually situated in areas with less human influence, whereas beaches of the inhabited islands and coasts are not frequented to the same degree^11,12^.

Harbour seals from the Wadden Sea undertake regular offshore trips to the North Sea, which can cover several hundred kilometres and last for several days to weeks^13–16^. While it was previously thought that these trips target areas with increased prey availability enabling intensive foraging^15^, recent research using high resolution movement sensors suggests that feeding rates do not differ considerably between transit and offshore periods^13^. This demonstrates that harbour seals access broadly distributed prey and have a constant high rate of food intake^13^, resulting in an increased flexibility in exploiting prey resources and leading to a possibly reduced dependency on local high prey density areas.

Several studies from different regions of the Wadden Sea have assessed the spatial usage of harbour seals in the Wadden Sea and North Sea^13,14,17,18^. However, harbour seals also enter adjacent estuaries^19^. A recent study conducting monthly aerial surveys in the inner and outer Elbe estuary in Germany demonstrated that harbour seals are present in this area throughout the whole year with the number of counted individuals ranging between 65 and 531^20^. The estuarine section of the Elbe entering into the Wadden Sea near the city of Cuxhaven, Germany, is characterised by brackish and freshwater environments and influenced by the tides of the North Sea, and there are sandbanks and river banks available for haul out up to Hamburg harbour^21,22^.

The Elbe, originating in the Czech Republic, is one of the major rivers in Europe with regard to length and related catchment area^23^. While increasing effort to protect the different habitats within the Elbe estuary has led to ecological mitigations in some parts, the area still needs to be considered as a major hot spot of anthropogenic activity with recreational and commercial shipping (with 8719 seagoing commercial vessels in 2016 visiting Hamburg harbour) and large-scale sediment dredging and dumping operations taking place on an almost constant level^22,24,25^. In order to maintain the required depth for large ships within the harbour of Hamburg, 3–9 million m^3^ of sediment needs to be extracted, while for maintenance of the channel from the harbour up to the mouth, another 12 million m^3^ are extracted annually^22^. Furthermore, pollutant levels are still considered high enough to affect the environment and respective communities^26^. In this context, previous studies have already shown that harbour seals inhabiting the Elbe estuary have significantly higher concentrations of several trace metals in their blood compared to animals from more distant sites in the Wadden Sea or Helgoland^27,28^. These developments have sparked considerable conflicts between the conservation of species and the anthropogenically induced alteration of the area^29,30^.

To date, no study has ever assessed the movements and spatial usage of harbour seals in the Elbe estuary. Therefore, we equipped harbour seals with biotelemetry devices to record GPS locations and basic behavioural data over the course of several months. In this study, we aim to assess the trip characteristics and haul-out behaviour of harbour seals in the Elbe. Furthermore, we computed home ranges to demonstrate the spatial usage of seals in this riverine environment. These analyses enable a comparison with data collected on harbour seals from the adjacent Wadden Sea and beyond. Furthermore, these data may also aid decision-makers during planning processes of anthropogenic activities as well as for the development of appropriate management regulations.

## Material and methods

### Study area

**Figure 1:**
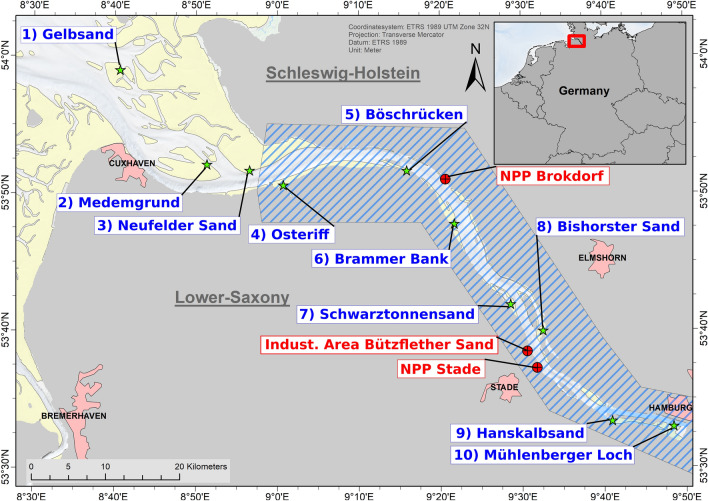
Map of the research area as part of the Elbe estuary. Green stars indicate known haul outs based on the data presented in Taupp^20^ which were considered as locations for catching harbour seals. Red markers indicate locations of industrial sites, including the two nuclear power plants (NPP) Brokdorf and Stade. The blue hatched area represents the area considered as inner estuary. The map was created using ArcGIS Vers. 10.6 73.

Locations considered for catching seals spanned from the haul out “Mühlenberger Loch” on the borders of Hamburg harbour in the south-east to the outer sandbank “Gelbsand” which is located north of Cuxhaven in the outer Elbe estuary (Fig. 1). For the purpose of this study, we differentiate between the outer and inner estuary, with the latter being defined as the area up until the river mouth where the estuary widens considerably (see blue hatched area in Fig. 1). At Hamburg, the mean salinity is < 0.5 PSU, and at Cuxhaven it reaches ~ 20 PSU^21^. The mean tidal range in the estuary is ~ 3 m^31^.

All haul outs showed seals present during aerial surveys, with the haul outs “Medemgrund”, “Neufelder Sand” and “Brammer Bank” being the most frequented ones^20^.

The habitat available to the seals within the Elbe estuary consists of the river channel representing the deepest part with around 14.5 m depth, bordered by regularly submerged tidal areas as well as river banks which in part consist of stretches with bank reinforcement, sandy beaches as well as reed belts. Furthermore, some smaller islands, mostly with restricted access to the public, are to be found in this part of the Elbe. The estuary width ranges between 2.5 km at the “Mühlenberger Loch” and 17.5 km in the area of the river mouth^22^. Besides these natural areas, several anthropogenic structures like harbours, locks and watergates are present throughout the study area. The nuclear power plant (NPP) “Brokdorf” is situated across the river from the haul out “Böschrücken” and pumps cooling water through an outlet into the Elbe (Fig. 1). The NPP “Stade” situated further south was shut down in 2003 but is still in the decommissioning phase.

### Seal catching and tagging

Catches were conducted between November 2019 and April 2021 using two small boats (ca. 6 m in length), powered by an outboard motor with 90 hp and 60 hp, respectively. In areas with low current speeds, catching took place based on the method described in Jeffries et al.^32^ using a net of 30 m in length and 5 m in height with a mesh size of 10 cm deployed between the two boats. The bottom line of the net consisted of a lead line that kept the lower part of the net as low as possible. The top line of the net had floats attached at regular intervals. In this combination, the net positions itself well in the water column and animals fleeing in the water can be caught. Where stronger currents were present, a group of hauled out harbour seals was approached with the two boats directly and animals were caught using pole- and hoop nets as the use of the large net was not practical.

After a thorough veterinary adspection, all animals were equipped with an SMRU GPS Phone Tag^33,34^ programmed to transmit data at 6 h intervals. The GPS interval was set to 10 min to reveal fine-scale movements in the river. The tag registered dive depth using a pressure sensor and haul out events via a wet/dry sensor^33^. The latter was fine-tuned by the manufacturer to also function in fresh water. During the instrumentation, the seal’s pelage was first dried on the back between the shoulder blades with a towel and then degreased with an acetone-infused cloth. Then, the biotelemetry device was glued to the pelage with a conventional high viscosity ethyl-based general purpose instant adhesive (Henkel LOCTITE 422). After the glue had cured and the device was properly attached (usually after 5–7 min), the animal was instantly released back into the wild. The biotelemetry devices remain on the animal until the following annual moult between July and September and fall off without leaving any remains when the hair is lost.

### Data processing and analysis

All data processing, analysis and, if not stated otherwise, visualisation were conducted in *R* version *3.6.2*^35^ using *R Studio 2021.9.0.351* for Windows^36^. Graphs were created using the package *ggplot2*^37^.

Prior to any analysis, the recorded GPS positions were filtered using the *SDLfilter* package. For this purpose, first the function “dupfilter”^38^ was used in order to filter out any temporal or spatial duplicates. Furthermore, the maximum linear speed between two consecutive positions using the function “vmax" as well as the maximum one-way linear speed of a loop trip using the function “vmaxlp” were calculated with the measure of position quality qi set to 4 (representing the number of detected satellites). The resulting values (vmax: 8.01 km/h, vmaxlp: 5.03 km/h) were then used in the function “ddfilter”^39^ to remove remaining erroneous positions.

Especially within the inner estuary, several haul out events showed unrealistic locations (e.g. in the centre of the shipping lane). Although the manufacturer had fine-tuned the conductivity sensor to ensure reliable performance also in water with low salinity, some incorrect haul out events in freshwater were recorded. As these incorrect haul-out events could not be reliably separated from plausible events based on haul out duration, previous dive depths, location accuracy and number of satellites, we chose to exclude any haul out location with a bathymetric depth of more than 3.5 m (9.5% of the haul out locations, see Figure A. 7 in the Supplementary materials). This resulted in a few potentially correct haul outs recorded in the outer estuary being also excluded due to low position accuracy. However, the majority of the exclusions were considered appropriate, based on a detailed visual cross-check of the excluded data and the additional information given in the haul out data (e.g. duration of haul out lasting only several seconds).

Based on the start and end times, we calculated the duration of each single haul out event, hereafter termed **single haul out duration**. Furthermore, we computed the **summed haul out duration per day**.

Trips were defined as any movement between haul out events covering distances of ≥ 1 km. The 1 km limit was set in order to avoid overemphasizing short trips in the water that harbour seals often make close to haul out sites^40^. The **trip length** resembled the summed distances between each consecutive pair of locations between two haul out events and was calculated using the function “trackDistance” from the *trip* package^41,42^. The **distance covered per day** by the animals was calculated by adding up the distances between all consecutive pairs of positions recorded during one day, again using the function “trackDistance”. The **trip extent** described the maximum straight line distance during each trip measured from the location of the previous haul out and was calculated using the “spDistsN1” function of the *sp* package^43^.

The **trip duration** was defined as the length of the period between the first and last location after and before a haul out event.

For each seal, home ranges were inferred based on GPS locations received by using the biased random bridge kernel method implemented in the “BRB” function of the *adehabitatHR* package^44^. The diffusion coefficient was estimated using the “BRB.D” function of the same package. The smoothing parameter (hmin) was set to 200 m. This kernel density estimation method is particularly suitable for telemetry data, as it treats the animals’ locations as originating from a movement process^45,46^. From the resulting utilisation distributions (UDs), we derived 50% and 95% home ranges. In order to give a better approximation of the spatial area actually used by the seals, the calculated home range area represented the calculated area of the UD after deducting area overlapping with land.

In order to investigate differences in trip characteristics and haul out behaviour between different biological periods, the data were separated into “Pupping Season” and “non-pupping season”. The pupping season was defined as the period between 1 June and 31 July, following Brasseur et al.^5^. The non-pupping season was defined as all remaining days outside this period.

Bathymetry data and information on geo-morphological zones in the Elbe estuary were provided by the Federal Institute of Hydrology^47^. Bathymetry data for the North Sea were accessed from the EMODnet Bathymetry project^48^.

### Statistical analysis

Linear mixed effect models (LMMs) were used to investigate the effect of sex (male/female) and season (non-pupping season/pupping season) on the six response variables: trip length, trip extent, trip duration, distance travelled per day, single haul out duration and summed haul out duration per day. Initial models included both explanatory variables and their interaction as fixed effects. Seal identities were included as random effects to account for individual variability among the animals. Assumptions of LMMs that residuals were normally distributed and homoscedastic were assessed using Q-Q plots as well as plots of fitted values vs. residuals. In this context, all response variables were either log-transformed (trip length, trip extent, trip duration, single haul out duration) or square-root-transformed (distance travelled per day, summed haul out duration per day) to ensure normality and homoscedasticy of residuals^49^. Independence was validated by plotting the residuals vs. the explanatory variables as well as by checking for temporal autocorrelation using the “acf” function in R. As the response variables displayed considerable temporal autocorrelation, an auto-regressive model of the order 1 (AR1) was added to the LMMs effectively eliminating the autocorrelation.

Model selection followed the steps recommended by Zuur et al.^49^. Each explanatory variable was alternately dropped from the full model and then this reduced model was compared to the full model by means of likelihood ratio tests (LRTs). The LRT compares two nested models based on the ratio of their likelihoods and provides a likelihood ratio statistic with an associated p-value, which is used for model inference. We first tested whether the interaction between the two explanatory variables (sex and season) should be retained by comparing it to a model without interaction using LRTs. If it could be dropped, we alternately excluded the two fixed effects ‘sex’ and ‘season’ and compared these models to the full model without interaction. The outcomes of the model selection based on LRTs (likelihood ratio statistics and *p*-values) are provided in the Results section. In the selection process, both the full and the reduced models were fitted with maximum likelihood approximation to assess the optimal fixed effect structure, while the most parsimonious model was refitted in the end using restricted maximum likelihood^49,50^.

Statistical analyses were conducted in the R software^51^. All LMMs were fitted using the function “lme” and LRTs were performed using the function “anova.lme” (R package *nlme*; Pinheiro et al.^52^). Statistical significance was set at *p* ≤ 0.05. If not stated differently, values are presented as mean ± standard error (SE) as derived from the model predictions.

### Ethics approval

All catches, sampling and tagging were conducted in compliance of all national laws and following current best practice guidelines for the attachment of biotelemetry devices and were approved by the responsible governmental ethics committees named hereafter for the specific catching sites. All these procedures were conducted under animal experiment ethical permits, with catches in the state of Hamburg approved under the permit V1305/591-00.33 of the former Hamburg Ministry of Health and Consumer Protection, catches in the state of Lower Saxony approved under the permit 33.9-42502-04-17/2562 and 33.12-42502-04-17/2562 of the Lower Saxony State Office for Consumer Protection and Food Safety-LAVES and catches in the state of Schleswig–Holstein approved under the permit V 241-64499/2018(11-2/19) of the Ministry of Energy, Agriculture, the Environment, Nature and Digitalization of Schleswig–Holstein. Additional permits for animal handling as well as for entering conservation areas were obtained from the local governmental hunting and nature conservation agencies of each respective county.

## Results

### Capture

In total, nine adult harbour seals were caught and tagged in the Elbe estuary. The deployment furthest into the estuary took place on the site “Hanskalbsand” and the most outer one at “Medemgrund” (Table 1, Fig. 1). Six of these animals were tagged in the inner part of the estuary, while three were tagged in the outer estuary near the river mouth.

**Table 1:** Details on harbour seals caught in the Elbe estuary and equipped with an SMRU GPS Phone Tag. Females = bold.

#	Tag ID	Capture site	Latitude	Longitude	Capture date	Length (cm)	Weight (kg)	Deployment duration (days)
**1**	**seal_1**	**Hanskalbsand**	**53.552246**	**9.692783**	**2019–11–29**	**184**	**-**	**172**
**2**	**seal_2**	**Brammer Bank**	**53.796518**	**9.363946**	**2020–11–06**	**161**	**50.5**	**268**
**3**	**seal_3**	**Böschrücken**	**53.865657**	**9.251415**	**2021–03–22**	**145**	**100.6**	**108**
4	seal_4	Medemgrund	53.872818	8.847082	2021–03–30	172	92.8	65
5	seal_5	Brammer Bank	53.796518	9.363946	2021–04–14	157	94.3	18
**6**	**seal_6**	**Brammer Bank**	**53.796518**	**9.363946**	**2021–04–14**	**172**	**81.5**	**89**
7	seal_7	Brammer Bank	53.796518	9.363946	2021–04–14	166	92.3	79
8	seal_8	Medemgrund	53.872818	8.847082	2021–04–15	183	102.9	59
**9**	**seal_9**	**Neufelder Sand**	**53.870564**	**8.989982**	**2021–04–15**	**171**	**97.5**	**87**

**Figure 2:**
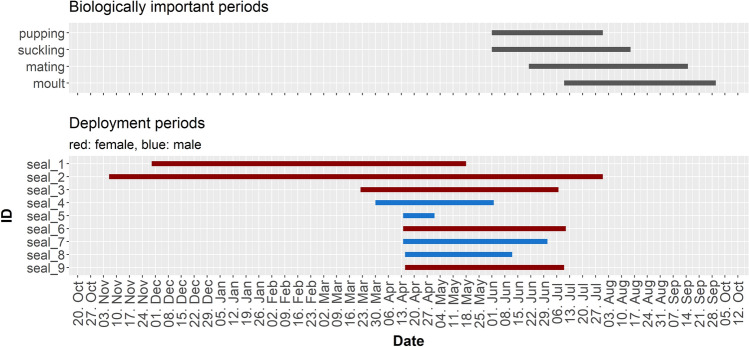
Top: Biologically important periods for harbour seals in the region. Bottom: Deployment periods throughout the year separated by ID and coloured by sex. Note that seal_1 was tagged in 2019 and seal_2 in 2020, while the other deployments took place in 2021; see Table 1.

The deployment of two animals started in November (one in 2019, one in 2020) and lasted throughout the winter, while the deployment of the other animals started during spring 2021 and lasted until the start of the moulting season (Table 1, Fig. 2).

### Spatial and temporal usage of the Elbe estuary and adjacent areas

**Figure 3:**
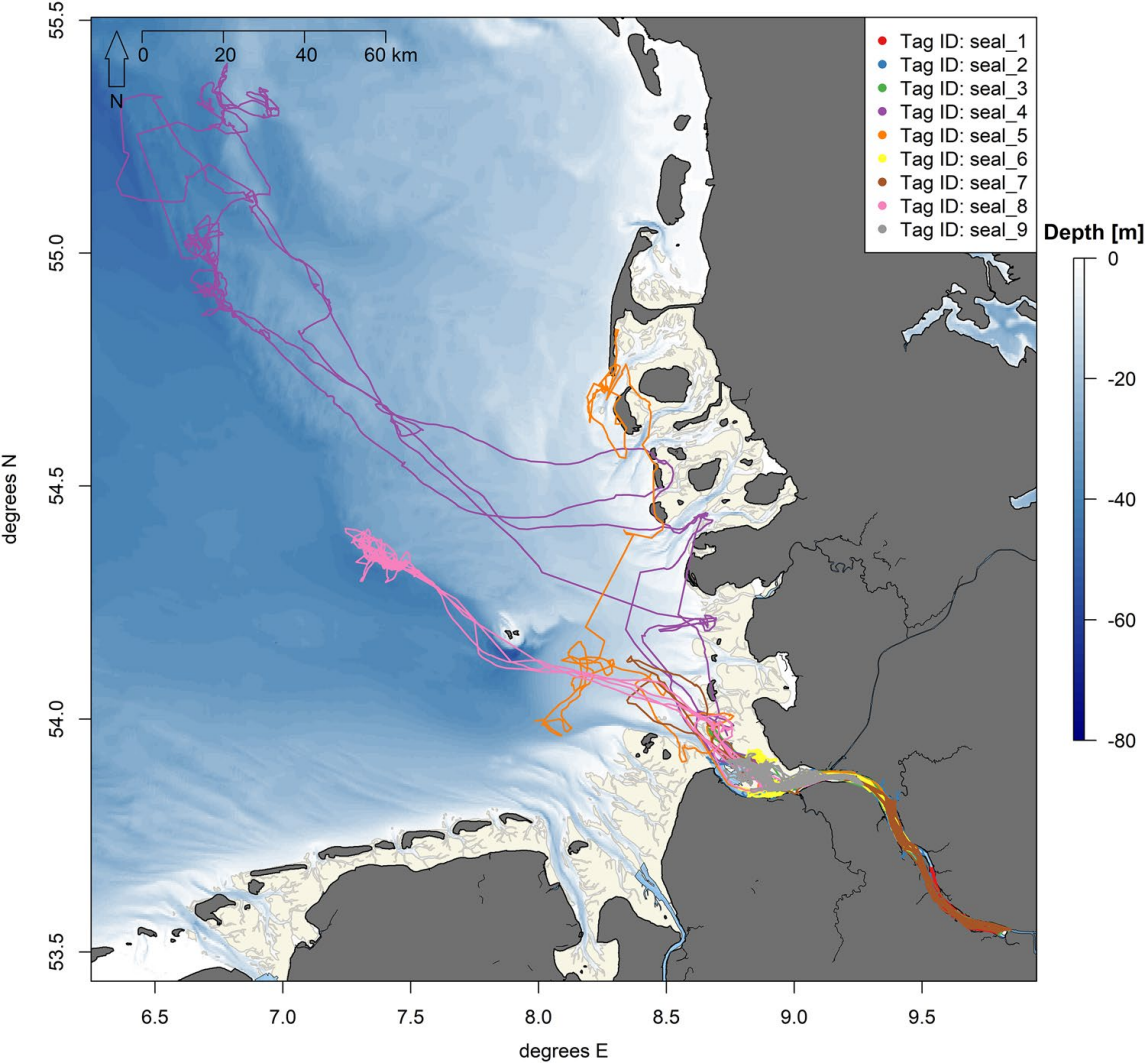
Map showing the recorded tracks of nine harbour seals tagged in the Elbe estuary. Tracks are colour-coded by tag ID. Visualisation was done in *R! 3.6.2*^35^ using *R Studio 2021.9.0.351* for Windows^36^ (https://cran.r-project.org/bin/windows/base/old/3.6.2/).

**Figure 4:**
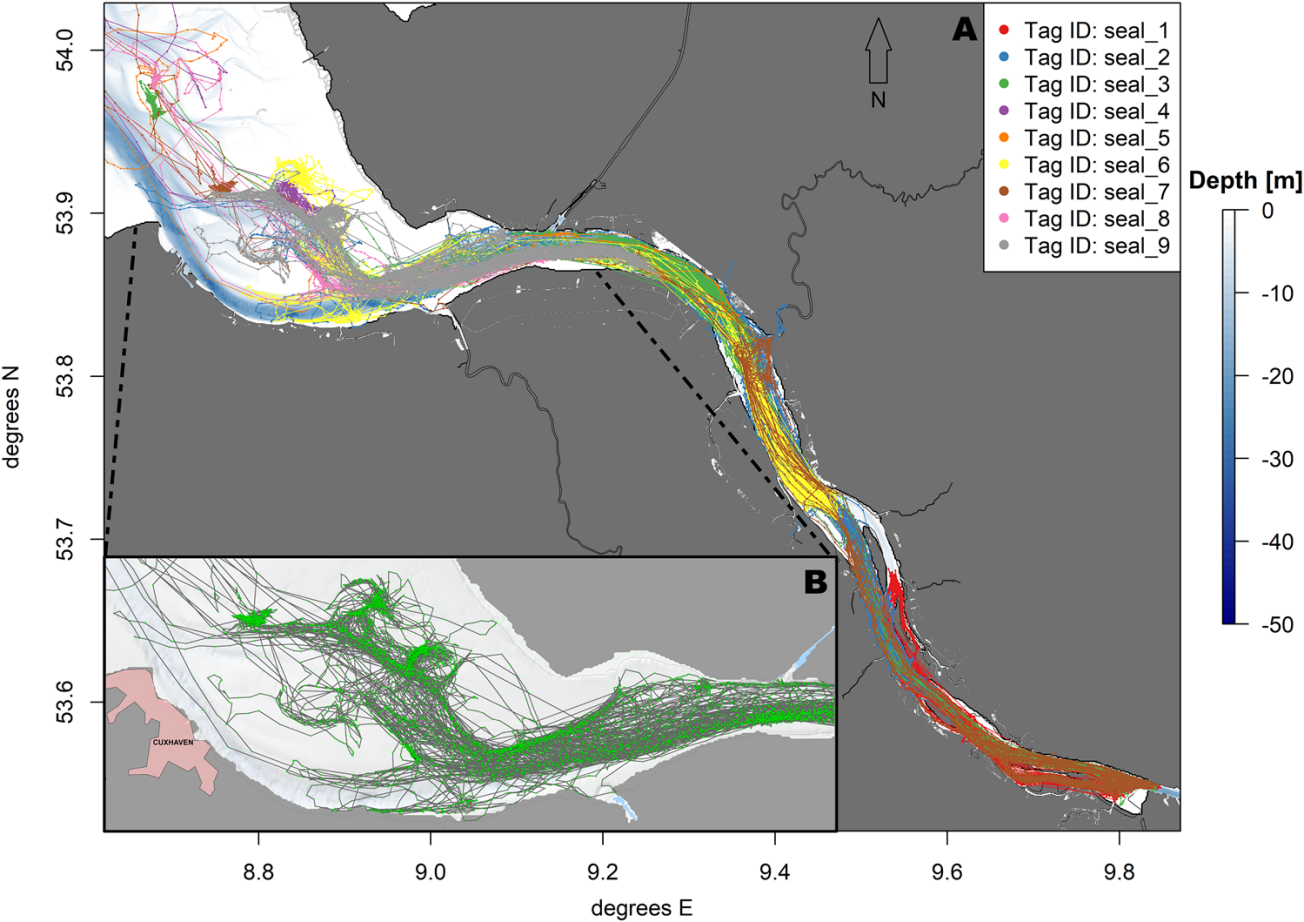
(**A**) Map with reduced extent showing the recorded tracks of nine harbour seals tagged in the Elbe estuary. Tracks are colour-coded by tag ID. (**B**) Focus map of the estuary near Cuxhaven. Green points: GPS locations, grey lines: tracks of the seals. Visualisation was done in *R! 3.6.2*^35^ using *R Studio 2021.9.0.351* for Windows^36^ (https://cran.r-project.org/bin/windows/base/old/3.6.2/).

During most of the deployment period, the animals stayed within an area spanning from the outer estuary near Cuxhaven (river kilometre 729) to the border of Hamburg harbour near Finkenwerder (river kilometre 631) (Figs. 3, 4A). While most animals stayed in the vicinity of their previous haul out site (trip extent outside pupping season: 3.5 ± 1.07 km, inside pupping season: 0.7 ± 1.11 km, Table 2, Figure A. 2 in the Supplementary materials), three male individuals (marked with * in Table 2) performed long distance trips (max. trip extent: 176.4 km) to the North Sea, with two animals targeting areas on the edge of the North Frisian shelf (Fig. 3). Also, one female (seal_1) constantly performed medium-length trips, resulting in a higher-than-average median trip extent of 15 km (Table 2, Figure A. 2 in the Supplementary materials). None of the animals moved more than a few kilometres to the west to the Wadden Sea in Lower-Saxony. One seal (seal_2) also moved a few kilometres into the tributary river Stör, which flows to the Elbe estuary on the opposite riverside of the haul out “Brammer Bank” (Fig. 1).

**Table 2:** Median/maximum trip extent, sizes of home ranges and proportion of the deployment duration spent in haul out or in the water for each individual for the full recorded period. Individuals that performed long distance trips are marked with *. Females = bold.

Tag ID	Median/max trip extent (km)	Size of home range 50%/95% (km^2^)	Proportion of time spent in haul out/in water (%)
**seal_1**	**15/24**	**13/37**	**20/80**
**seal_2**	**5/36**	**18/110**	**30/70**
**seal_3**	**1.8/54**	**16/125**	**21/79**
seal_4*	4/176	72/892	12/88
seal_5*	12/53	50/285	15/85
**seal_6**	**4/35**	**14/100**	**22/78**
seal_7	7/75	12/135	24/76
seal_8*	14/123	22/285	15/85
**seal_9**	**4/26**	**17/87**	**23/77**

Besides the main channel, harbour seals also moved through tributaries of the river and used them for hauling out. While the main channel of the estuary including the shipping channel was used by the seals within the inner estuary, as soon as they reached the river mouth, the majority of tracks left the part near the shipping channel into the tidal channel Medemrinne (Fig. 4B).

Seal_2 and seal_3 both extensively used the area around the cooling water outlet of the NPP “Brokdorf” situated across the river from the haul out “Böschrücken” (Fig. 1). There was a strong aggregation of locations and dives of both animals close to this outlet. While seal_2 regularly visited this spot over consecutive weeks at a time while remaining in the vicinity throughout (Figure A. 4 top in the Supplementary materials), seal_3 did not spend longer periods in front of the outlet but rather kept on visiting the site on short occasions throughout the whole deployment period (Figure A. 4 bottom in the Supplementary materials).

While the seals’ movements showed no limitation towards and beyond the outer estuary, no animal moved past a distinctive point just at the entrance of the Hamburg harbour in the inner estuary (Figure A. 5 in the Supplementary materials).

### Haul out behaviour

**Figure 5:**
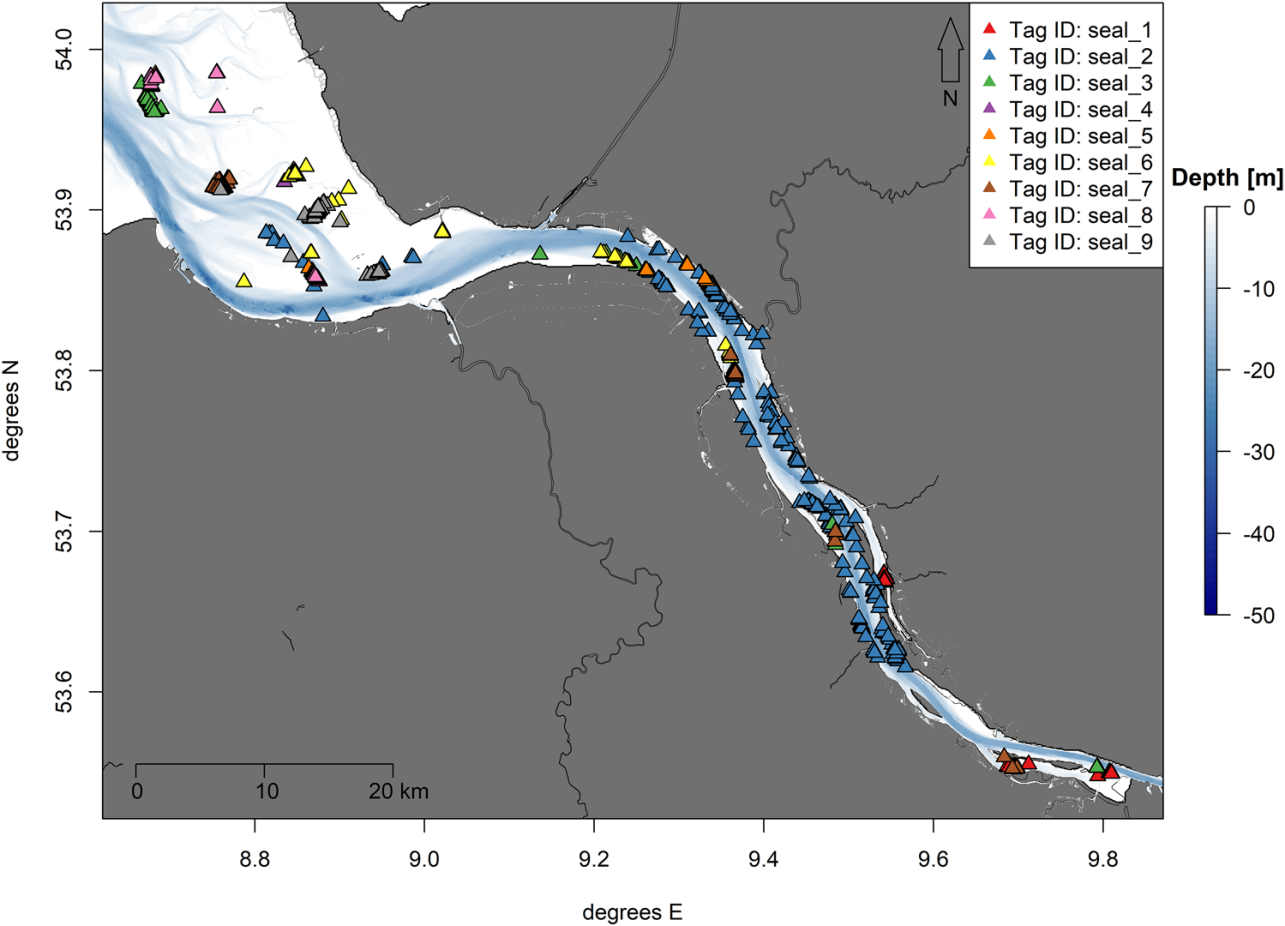
Map with reduced extent showing the recorded haul out sites of nine harbour seals tagged in the Elbe estuary. Haul outs are colour-coded by tag ID. Visualisation was done in *R! 3.6.2*^35^ using *R Studio 2021.9.0.351* for Windows^36^ (https://cran.r-project.org/bin/windows/base/old/3.6.2/).

The individuals that travelled into the outer estuary only used known haul out sites in the Wadden Sea, whereas three of the individuals within the inner estuary also chose sites previously unknown as haul outs (Fig. 5). Seal_2 showed the highest rate of haul outs outside known sites (blue triangles in Fig. 5), with the chosen spots spreading over most of the deployment area.

**Figure 6:**
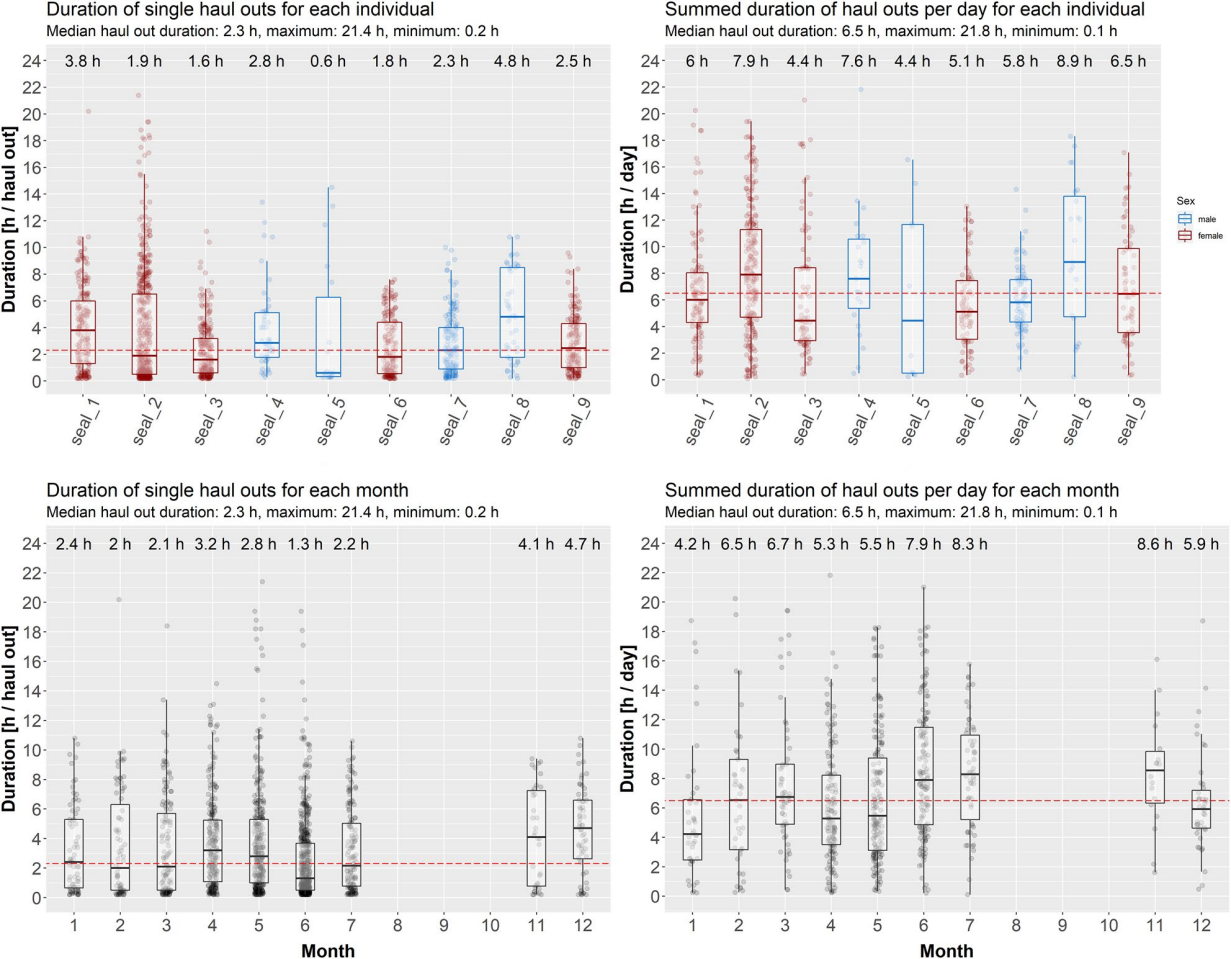
Box plots showing the duration of haul outs for each individual (top row) and each month of the year (bottom row) comparing the duration of single haul out events (left column) with cumulative haul out duration for each day (right column). Boxes represent the 25th and 75th percentiles, the bar the median and whiskers extend from the hinge to the largest or smallest value but no further than 1.5 * the inter-quartile range. The x-axis shows the seal ID.

Overall, the animals spent around 21% (median, range: 12–30%) of the deployment period hauled out (Table 2). Duration of single haul out events showed differences between the pupping and non-pupping season (likelihood ratio statistic: 18.89, *p* < 0.0001). Haul out events were significantly longer during the non-pupping season (2.0 ± 1.09 h) than compared to the pupping season (1.5 ± 1.07 h). No significant difference was detected between the sexes (likelihood ratio statistic: 0.29, *p* = 0.59). For the summed haul out duration per day, the interaction between season and sex was significant (likelihood ratio statistic: 8.17, *p* = 0.004). The daily haul out duration for females added up to 5.2 ± 0.01 h outside the pupping season, and 8.2 ± 0.01 h during the pupping season and for males 6.4 ± 0.04 h outside the pupping season and 6.7 ± 0.03 h during the pupping season (Fig. 6). Even though the duration of single haul out events was reduced during the pupping season, the cumulative daily haul out duration actually increased during this period. Especially females spent more time hauled out per day (increase of 57.7%) than males (increase of 4.7%) during the pupping season.

### Trip characteristics

The animals covered a wide range of distances from 2 to 109 km per day and a significant interaction was found between season and sex (likelihood ratio statistic: 17.2, *p* < 0.0001). On average, distances covered per day per individual amounted to 32.4 ± 0.05 km/day outside the pupping season and 15.2 ± 0.04 km/day during the pupping season for females, and for males to 28.1 ± 0.12 km/day outside the pupping season and 28.2 ± 0.18 km/day during the pupping season (also see Figure A. 3 in the Supplementary materials).

Likewise, a significant interaction between season and sex was detected for the trip lengths (likelihood ratio statistic: 4.73, *p* = 0.03) and durations (likelihood ratio statistic: 4.38, *p* = 0.04). The length of single trips conducted was 9.0 ± 1.12 km outside the pupping season and reduced to 1.6 ± 1.14 km during the pupping season for females and 7.0 ± 1.24 km outside the pupping season, and 2.5 ± 1.37 km during the pupping season for males. Single trips of females lasted 6.6 ± 1.09 h outside the pupping season and 2.1 ± 1.12 h during the pupping season, while single trips of males were on average 4.9 ± 1.19 h outside the pupping season and 2.6 ± 1.30 h during the pupping season.

**Figure 7:**
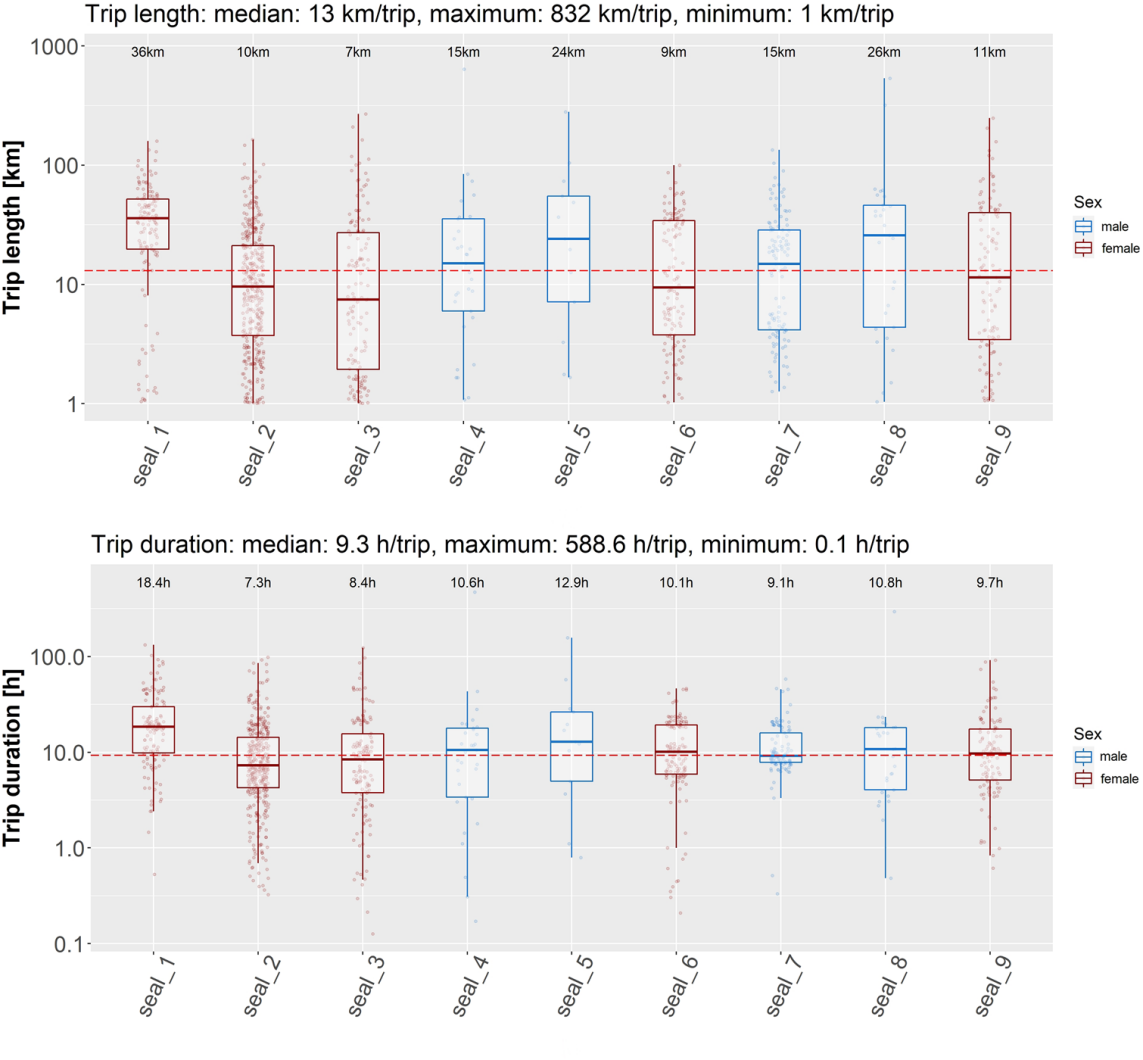
Length (top) and duration (bottom) of trips for each individual coloured by sex. The data values shown on the y-axis were log transformed to account for outliers present in the data. Median values of each individual are given at the upper end of each boxplot. Overall maximum and minimum values are shown in the plot title. Boxes represent the 25th and 75th percentiles, the bar the median and whiskers extend from the hinge to the largest or smallest value but no further than 1.5 * the inter-quartile range. The x-axis shows the seal ID.

There were few trips with comparably long durations and lengths (e.g. trip length of 832 km or duration of 588.6 h, Fig. 7), which represent offshore trips, in this case targeting the North Frisian shelf area, a behaviour commonly observed in animals from the Wadden Sea^13,18^.

However, for the trip extent, no difference was found between the sexes (likelihood ratio statistic: 0.1, *p* = 0.75), whereas comparing the two seasons revealed a significant difference (likelihood ratio statistic: 157.93, *p* < 0.0001) with significantly reduced trip extents during the pupping season. On average the trip extent outside the pupping season was 3.5 ± 1.07 km and reduced to 0.7 ± 1.11 km during the pupping season.

In summary, the seals performed much shorter (reduction in trip duration of females: 68.2%, males: 46.9%) and less distant trips, both in terms of length (reduction in trip length of females: 82.2%, males: 64.3%) and extent (reduction in trip extent of 80.0%), during the pupping season compared to the non-pupping season.

### Home ranges

Despite the similarity in average trip length and duration between individuals, the size of the home range inferred from the total deployment period showed a distinct interindividual difference such that the three male individuals, which covered the greatest distances, also had the largest home range sizes (Table 2).

The median size of the home range calculated for all females was 16.3 km^2^ (HR 50%) and 99.5 km^2^ (HR 95%), while for all males, the median size of the home range reached 36.1 km^2^ (HR 50%) and 284.9 km^2^ (HR 95%), in both cases more than double the females’ home range size (Table 2).

**Figure 8:**
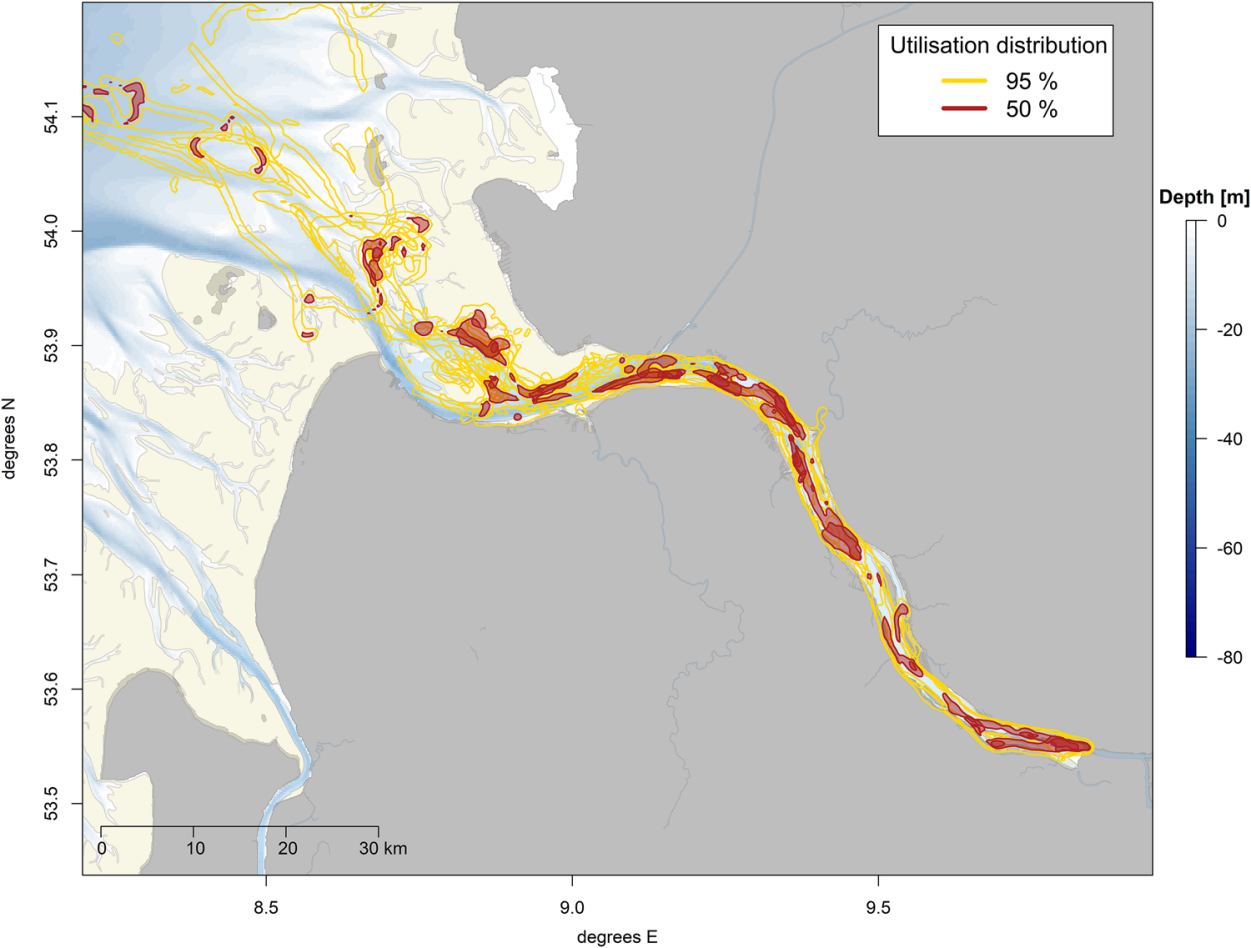
Map showing the overlayed utilisation densities (50%: red, 95%: yellow) of all tagged animals. While the 95% utilisation distribution resembles the overall home range, the 50% utilisation distribution can be regarded as the core area of each seal. Visualisation was done in *R! 3.6.2*^35^ using *R Studio 2021.9.0.351* for Windows^36^ (https://cran.r-project.org/bin/windows/base/old/3.6.2/).

When overlaying the individual home ranges, it becomes apparent that major parts of the inner estuary were utilised and core areas of multiple individuals overlapped. Especially the area between the haul out sites “Böschrücken” and “Schwarztonnensand”, but also the area just close to the entrance of Hamburg harbour were used regularly (Fig. 8). In the outer estuary, the areas around the haul out sites “Neufelder Sand” and “Medemgrund”, extending into the tidal channel Medemrinne in the North, were intensely utilised as well as the area around the haul out site “Gelbsand” (Fig. 8).

**Figure 9:**
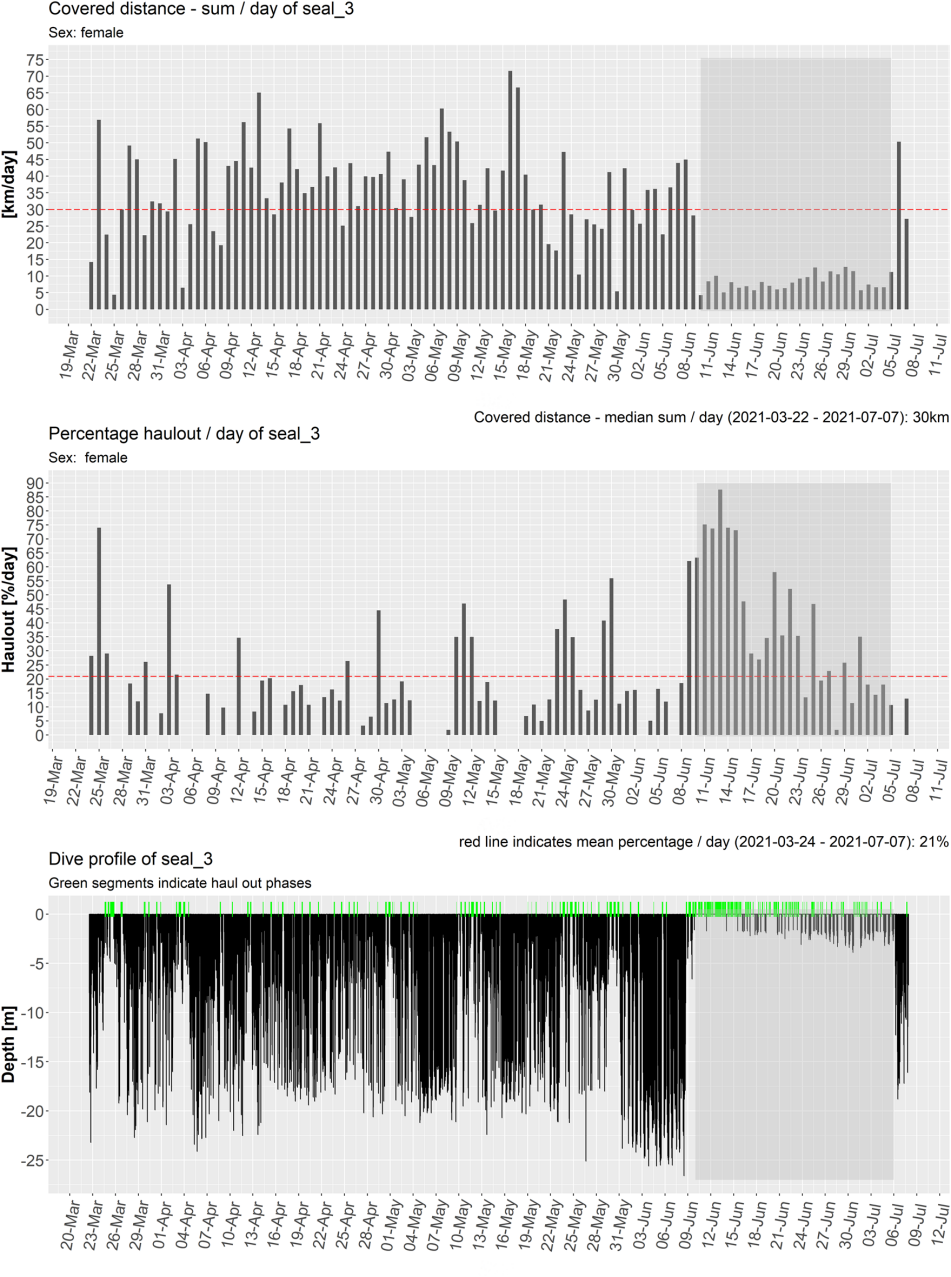
Bar plots showing the covered distance per day (top), the proportion of the day spent in haul out (middle) and the dive depth reached as well as haul out as green bars (bottom) for the female seal_3 as one example. Areas shaded in darker grey indicate the period of behavioural change indicative for the reproductive period.

A clear change in behaviour was observed during the months June to July, which was visible not only in the trip characteristics and haul out behaviour but also concerned the home range and diving behaviour of four seals (seal_3, seal_6, seal_7, seal_9). For example, three of four females tagged during this time showed a distinct change in movement patterns by abruptly reducing the distance covered per day, reducing the maximum dive depth as well as increasing the haul out rate (Fig. 9, Table 3). We hypothesise that this change in behaviour is indicative of parturition and lactation of a pup.

**Table 3:** Table showing the three female (bold) and one male individual exhibiting a shift in behaviour during the reproductive period. Presumed start and stop dates of the behavioural change were inferred from the behavioural data of each animal.

ID	Start date	Stop date	Duration (days)	Median distance travelled per day outside/inside presumed reproductive period (km)	Percentage haul out during a day outside/inside presumed reproductive period (%)	Size of home range (95%) outside/inside presumed reproductive period (km^y^)
**seal_3**	**2021–06–10**	**2021–07–05**	**25**	**37/8**	**12/38**	**127/2**
**seal_6**	**2021–06–13**	**2021–07–07**	**24**	**36/11**	**12/35**	**97/9**
**seal_9**	**2021–06–09**	**2021–07–10**	**31**	**49/9**	**17/36**	**88/16**
seal_7	2021–06–13	2021–07–01	18	36/9	23/27	140/3

**Figure 10:**
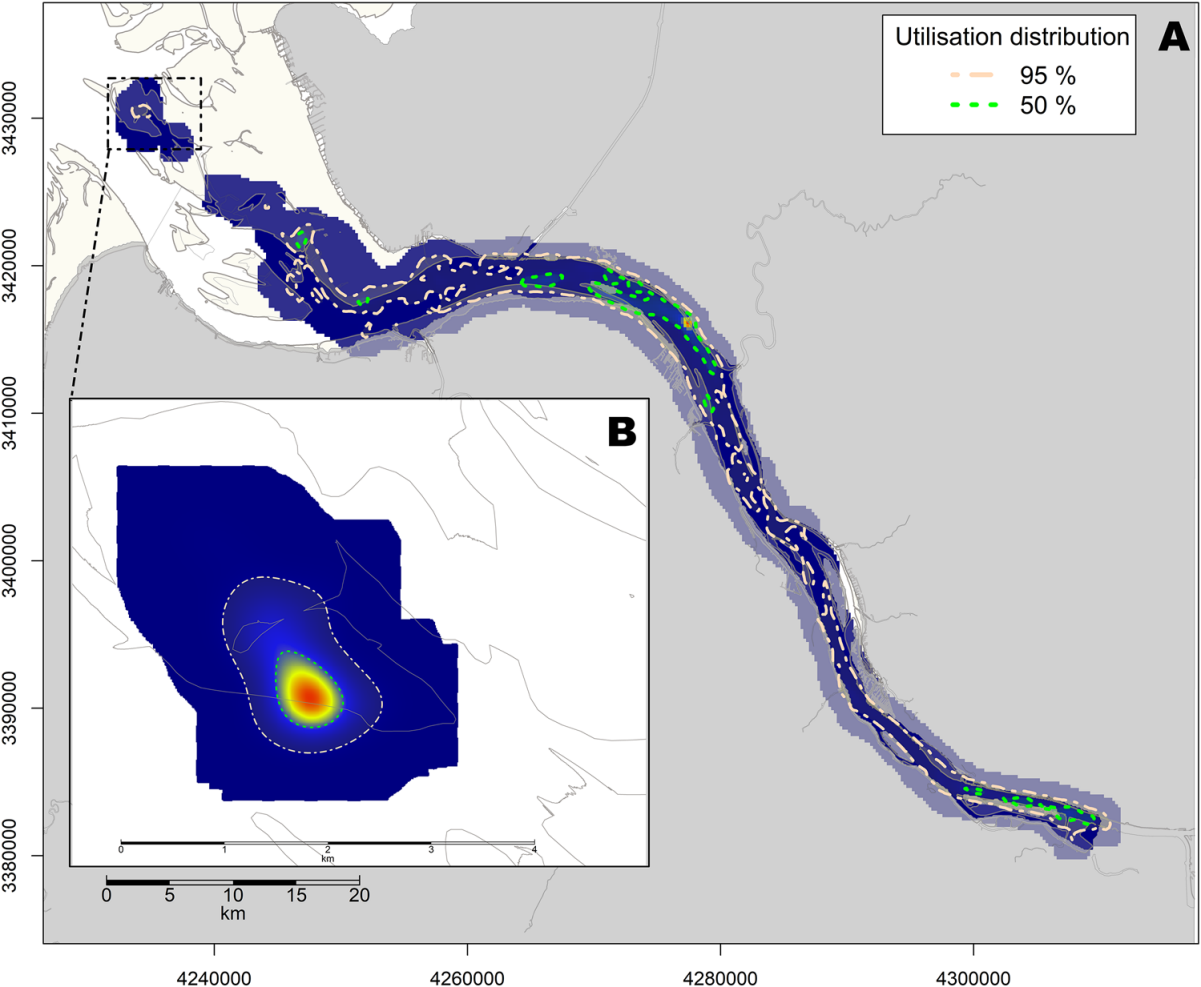
Home range inferred from the calculated utilisation distribution with the large map showing the home range for the period outside the presumed reproductive period and the small map showing the home range during the presumed reproductive period for female seal_3. 50% HR: green dashed line, 95% HR: beige dashed line. Light yellow areas represent intertidal habitat. Visualisation was done in *R! 3.6.2*^35^ using *R Studio 2021.9.0.351* for Windows^36^ (https://cran.r-project.org/bin/windows/base/old/3.6.2/).

In order to specifically depict this change in behaviour of the four seals, utilisation distributions and home ranges were calculated separately for the time before and during the presumed reproductive period. By reducing their distances travelled, the four animals showing this behaviour also considerably reduced their home ranges during the presumed reproductive period (Median HR 95%, outside: 112.1 km^2^, inside: 5.8 km^2^; median HR 50%, outside: 17.3 km^2^, inside: 0.5 km^2^, Fig. 10, Table 3).

Interestingly, also one of the male seals (seal_7) showed such a shift in behaviour. This individual reduced the distance covered per day from an average of 36 km to between 5 and 10 km, while at the same time not considerably increasing the amount of time spent in haul out (Table 3, Figure A. 6 in the Supplementary materials).

## Discussion

Along the North Sea coast of continental Europe and the UK, the harbour seal is an abundant predator and regarded as a key species in this ecosystem^3,14^. Previous studies have assessed the movement and habitat utilisation of harbour seals within the North Sea and adjacent coastal waters^13,15,53,54^. While there have been sightings of harbour seals in rivers and estuaries^19,20^, no studies have focused on how these seals utilise such brackish and fresh water environments adjacent to the North Sea. Despite the limitations resulting from the low sample size (e.g. data not covering all seasons), the results of this study provide a first insight into the movements of nine harbour seals equipped with biotelemetry devices within the Elbe estuary.

### Trip extent

In comparison to their fully marine living conspecifics, harbour seals from the Elbe estuary showed different movement patterns. Whereas previous studies have shown that harbour seals in the Wadden Sea and adjacent waters often undertake trips targeting offshore waters with distances to the colony exceeding 100 km^13,15,17,55^, the seals tagged in the Elbe estuary usually stayed close (average trip extent outside the pupping season: 3.5 km, max: 176.4 km, Table 2) to their previous haul out location. Yet, there were interindividual differences, with especially the male individuals conducting trips further away, either to offshore foraging areas or northern haul out sites in the Wadden Sea (Table 2, Fig. 3). This pattern is consistent with harbour seals from the inner Moray Firth, Scotland, where female seals have been shown to extend their foraging trips to an average of 14.9 km and males to an average of 25.5 km^40^. Nevertheless, the difference between sexes in this study (Table 2) is not statistically significant. In contrast to the results from the present study, but comparable to the results from Scotland, adult harbour seals in the Kattegat, Denmark conducted foraging trips to a maximum distance of 20 km away from the tagging site^56^.

### Trip length and duration

Similar to the trip extents, the median length of the trips was also well below the range described in other studies. For instance, comparisons of trip lengths from seals in the UK revealed slight differences between sexes, with 30.7 km^57^ and 30.1 km^40^ for females and 36.6 km^40^ and 61.1 km^57^ for males, respectively. However, these two studies showed a large variability between individuals and between tagging areas. This is also in accordance with the present study with trip lengths ranging from the set minimum of 1 km to a maximum of 832 km (Fig. 7). In our study, except for a few single longer trips, on average, male individuals covered significantly shorter distances during their trips than in comparison to the females during and outside the reproductive season while the trip duration of males was only shorter compared to that of females outside the pupping season and slightly higher during the pupping season. This result is in contrast to results from a large-scale study in Scotland where outside the pupping season males conducted trips with longer durations and distances covered in comparison to the females^57^. However, in comparison to the Scottish study, the data analysed in our study included relatively few animals.

Furthermore, the average trip durations during the respective seasons found in this study was notably shorter compared to other studies where average harbour seal trips lasted for up to 66.4 h^58^ or even 4.5 days^57^. The lower duration as well as length of harbour seal trips in the Elbe estuary might be the result of the confined space the animals can utilise in combination with various haul out sites in close proximity that can easily be accessed. Such conditions might potentially encourage the seal to haul out more often as opposed to a seal conducting longer offshore trips where suitable habitat for hauling out is not available. This could be of advantage as it has been shown that harbour seals increase the time spent resting at sea over the duration of a trip, indicating that resting at sea is not as energetically profitable as resting on land, thus resulting in the need to return from offshore waters to the haul out^13^.

In this study, movement patterns within the estuary varied among individuals. Some individuals (e.g. seal_1) utilised a very limited area in a routine fashion always targeting the same areas during trips and also showing a high site fidelity towards specific haul out sites (e.g. seal_1 used three distinct haul out sites during a deployment duration of 172 days). Other individuals used confined areas for some days, including specific haul out sites within these areas, and then moved to another part of the estuary to show the same pattern but in the end utilised a larger proportion of the deployment area. Only on one occasion did an animal move from the southern part near Hamburg up to the river mouth or vice versa in a single trip.

### Home range

The differences in spatial usage between the present and marine focused studies become even more evident when comparing the sizes of the home ranges which were inferred from the calculated utilisation distribution using the biased random bridge kernel method^44^. A study by Tougaard et al.^55^ showed, for example, that harbour seals tagged in the Danish Wadden Sea utilised areas ranging from 319 km^2^ (minimum HR 50%) to 6457 km^2^ (maximum HR 50%). In contrast, seals from this study (for details, see Table 2) utilised an area ranging from 12 km^2^ (minimum HR 50%) to 72 km^2^ (maximum HR 50%).

Due to the different approaches used to calculate the utilised areas though, a comparison between home ranges should be treated with caution. While the calculation of the simple kernel utilisation distribution as used in Tougaard et al.^55^ only considers the positions of the relocations, the biased random bridge kernel method also considers the track most likely followed by the individual between consecutive relocations and therefore represents a suitable method for analysing movement data^46^.

However, as the results are on different scales of magnitude, there is a strong indication that the harbour seals from the Elbe estuary only use a fraction of the home range area in comparison to seals from other sites. Further studies, including, for example, data on prey availability or high-resolution foraging behaviour of the seals as well as comparable data from the nearby Wadden Sea would be needed in order to assess the mechanisms underlying this difference ^e.g.^^13^.

### Haul outs

The harbour seals in this study used 21% (overall median) of their deployment duration for hauling out and thus spent 79% of their time in the water. Three of the four males spent considerably less time in haul out (12%, 15%, and 15% respectively) as compared to the other individuals (Table 2). While the overall median was within the range reported for harbour seals in Scotland (22%) and in the Netherlands (20% for females), the median percentage males hauled out is below the 22% level reported previously^17,40^.

In the present study, the duration of haul outs per individual outside the pupping season amounted to an average of 5.2 h per day for females and 6.4 h per day for males. This is longer than the median (4.39 h) that has been reported for seals from Scotland^59^. Conversely, the average duration of single haul out events of harbour seals in the Elbe estuary outside the pupping season was 2.0 h and thus noticeably lower than the reported 4.77 h in the study by Cunningham et al.^59^. Whether this was due to an increased rate of disturbance resulting in more frequent but shorter haul outs or to the increased chance to haul out remains unclear. One aspect supporting the hypothesis that disturbance by land-based human activities is one potential cause is that despite a higher availability of tidally unaffected sites suitable for haul out, seals predominantly chose tidally dependent haul outs, such as tidal mudflats. While being sub-optimal with regard to the tidally limited resting time, these spots do offer the advantage of being less frequented by humans. In contrast to the other animals, one seal (seal_2) also regularly used three specific sandy beaches on riverbanks in the inner estuary as haul out. Although numerous beaches are present throughout the study area, all of the three beaches used lay in a confined area. This choice can give further indication that disturbance partly affects haul out behaviour, as these beaches are likely to be less frequented by humans, given their location in front of industrial sites such as the large industrial area *Bützflether Sand* or the shutdown NPP *Stade* (Fig. 1). In order to assess this in greater detail, more sophisticated biotelemetry devices are needed such as high resolution sound and movement tags which record fine-scale movements of the animals and ambient noise conditions simultaneously and hence enable a better evaluation of disturbance events^60^. For a first assessment, also an observer-based study comparing the different haul outs with regard to seal presence and human activity would be suitable.

### Pupping season

Several tags deployed in spring 2021 transmitted up until the pupping season of harbour seals between June and July of that year. This enabled us to assess the movement patterns and respective changes with regard to the period prior to the pupping season (e.g. Fig. 9).

Three of four tagged females showed distinct shifts in behaviour, with a considerable reduction in movement and diving and a respective increase in haul out duration and frequency (e.g. seal_3 showed a reduction of the 95% HR from 127 km^2^ prior to 2 km^2^ after the presumed birth of the pup; see Table 3). Just prior to this shift in behaviour, all seals left the inner estuary in order to target sandbanks within the outer estuary and remain in their vicinity thereafter. The behavioural change lasted between 24 and 31 days (Table 3) and, in cases where tag transmission operated long enough, a return to the baseline behaviour before the presumed pupping was observed. The duration of the behavioural change is consistent with reported lactation durations. For instance, harbour seal mothers in Scotland suckle their pups for an average of 20 days, with a maximum lactation duration of 32 days^61^. Thus, the behavioural shift observed in the three females from this study is likely explained with parturition and lactation of a pup.

Besides the three females, one male individual also showed such a distinct shift in behaviour. Yet, in contrast to the females, the male did not considerably increase the time spent hauled out (Figure A. 6 in the Supplementary materials). The male individual stayed in the vicinity of the sandbanks where the females likely gave birth to their pups. The male also showed a reduction in the 95% HR from 140 km^2^ before the reproductive season to 3 km^2^ during the reproductive season for an 18-day period (Table 3).

Following the statistical analysis of the haul out data, a significant interaction between sex and season was detected, with females increasing their summed daily haul out duration during the pupping season by 57.7%, whereas males only increased their summed daily haul out duration during the pupping season by 4.7%. Interestingly, while the daily summed duration of haul outs increased during the pupping season, the duration of single haul outs decreased by 25%. This pattern is likely to be the result of repeated but shorter haul outs throughout a day, as the animals constantly stay in the area enabling them to haul out as soon as sandbanks start to get exposed during ebb-tide. The statistical analysis showed that this was mainly true for females though. While both sexes reduced the duration of single haul outs by a quarter, males showed an increase in their summed daily haul out duration by 4.7% only, indicating that the frequency of haul out events did not increase as much as compared to the females.

Such a shift in behaviour has already previously been reported for female and male harbour seals in the Netherlands^17^, Germany^62^, Scotland^63,64^ as well as Alaska^65^. While females stay in the vicinity of the place at which they gave birth and take care of their pups during the suckling period, males remain close by and compete for females, e.g. by performing underwater vocal displays^63^. The reported behaviour indicates that the seals from the Elbe estuary also regularly reproduce, displaying the same behavioural shifts as reported from other areas, but also show a spatial shift from the inner to the outer estuary. This phenomenon is also supported by the results of the aerial surveys during which adults with pups were, with one exception, only sighted in the outer estuary^20^. As especially female harbour seals are regarded to be highly philopatric^56,57,66^, it is possible that, given the increasing abundance and thus density of seals in the Wadden Sea over the last decades, animals have moved out of these areas due to increased intraspecific competition. These animals may have occupied available habitats in the Elbe estuary but still return to their natal sites in the Wadden Sea for breeding. Similar directional movements prior to the pupping season have also been reported for harbour seals in the Netherlands, where females move to the German Wadden Sea to give birth and raise their pups, supporting the assumption of a high breeding site fidelity^17^.

### NPP Brokdorf

Two of the tagged seals showed an explicit preference for an area in front of the cooling water outlet of the NPP Brokdorf. While this area was passed many times, but never frequented by other individuals, seal_2 and seal_3 used the outlet extensively (Figure A. 4 in the Supplementary materials). Before being discharged into the river, the water is used for cooling purposes and is allowed to be up to 10 °C warmer than the surrounding water (pers. commun. Preussen Elektra GmbH). However, while research in other areas on the overall impact of cooling water outlets on fish eggs and larvae as well as on the fish species richness has revealed a tendency suggesting negative effects^67,68^, it was also shown that the abundance of specific species can be similar or even higher^69^. This seems to be especially valid for species like the European eels (*Anguilla anguilla*) which are known to tolerate also warmer waters^67^. Thus, some fish species are attracted by warm water effluents, but whether the increased temperature attracts fishes and in turn seals or attracts the seals themselves, cannot be assessed with the present data.

### Limited upstream movement

While the seals in this study showed no spatial limitations when leaving the Elbe estuary, further upstream seals did not pass a certain line on the boundary of Hamburg harbour. Three of the tagged seals were active in this area and all of them refrained from crossing a certain line that coincides with the position of an Acoustic Doppler Current Profiler (ADCP) measuring current velocity. This operates from one riverside to the other and emits 0.2 s long signals at a frequency of 28 kHz with a pulse rate of 4.1 s^70,71^. The signal frequency is within the range of best hearing sensitivity of harbour seals^72^. Research using similar acoustic signals has shown deterrence effects within this frequency range^73^. Moreover, seals may also avoid entering the harbour area due to elevated noise levels from ship traffic. Not only cargo ships, but also tugs, ferries, cruise ships and many pleasure boats move through the harbour. Nonetheless, harbour seals are occasionally sighted in the Hamburg harbour area, albeit in negligible numbers. Answering the question whether high noise levels are actually the cause of this movement barrier would require further research using acoustic recording tags deployed on seals.

## Conclusion

In order to assess the movement and spatial usage of harbour seals in the German Elbe estuary, SMRU GPS Phone Tags were deployed on five female and four male individuals, which collected a total of 945 days of data. The study demonstrates that seals inhabiting the estuary displayed different movement patterns opposed to harbour seals from other regions. While home range sizes, trip durations or lengths tended to be reduced, overall daily haul out rates were higher.

Furthermore, interindividual variability of behavioural and movement patterns was high. Several individuals developed specific strategies (e.g. exploiting the cooling water outlet or hauling out on beaches) demonstrating the high plasticity in behaviour of harbour seals in this dynamic environment. During the pupping season, seals from the Elbe displayed similar behavioural shifts as have been reported for other areas, by reducing the length, extent and duration of their trips as well as shortening the duration of single haul out events while increasing the summed daily duration of haul outs. Interestingly, all animals moved out of the inner estuary to the outer estuary prior to the pupping season, highlighting a possibly strong natal philopatry.

While the low sample size in this study only allows for a limited interpretation of the data, these first results indicate that the seals from the Elbe have adapted to living in this highly anthropogenically altered habitat. Yet, a larger set of data is needed along with detailed data recorded by high resolution multi-sensor tags in order to be able to assess movements and potential disturbances in greater detail.

## Supplementary Information

Supplementary Information.

## Data Availability

All data generated or analysed during this study are included in this published article [and its Supplementary information files].
