# Supplementary material for: Movements and spatial usage of harbour seals in the Elbe estuary in Germany
Source: Sci Rep. 2023 Apr 24;13:6630. doi: 10.1038/s41598-023-33594-1 (PMC10125962; doi:10.1038/s41598-023-33594-1)
Supplement: Supplementary file 1 — Supplementary Information. [file 41598_2023_33594_MOESM1_ESM.pdf]

Movements and spatial usage of harbour seals in the Elbe estuary in Germany

Authors:

Abbo van Neer, Dominik Nachtsheim, Ursula Siebert, Thomas Taupp

Supplementary materials

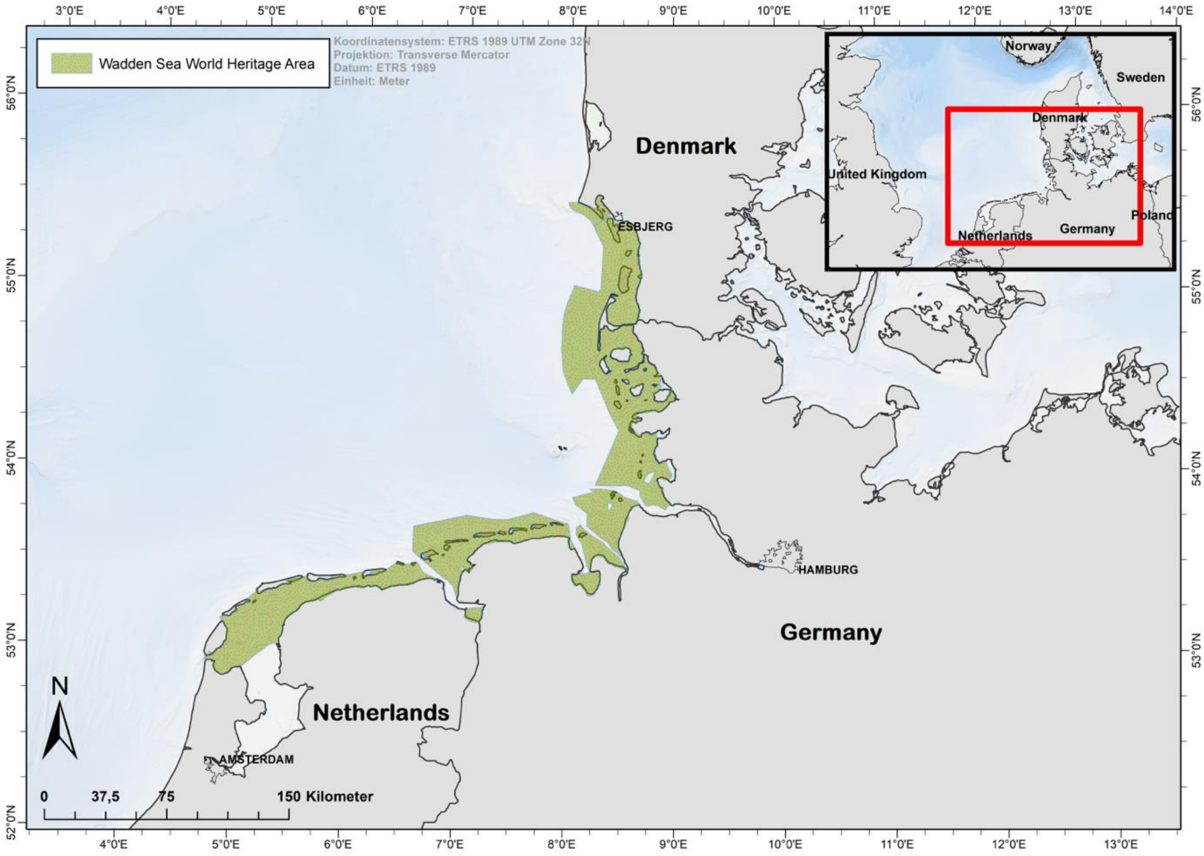

Figure A. 1: Overview map of the trilateral Wadden Sea area.

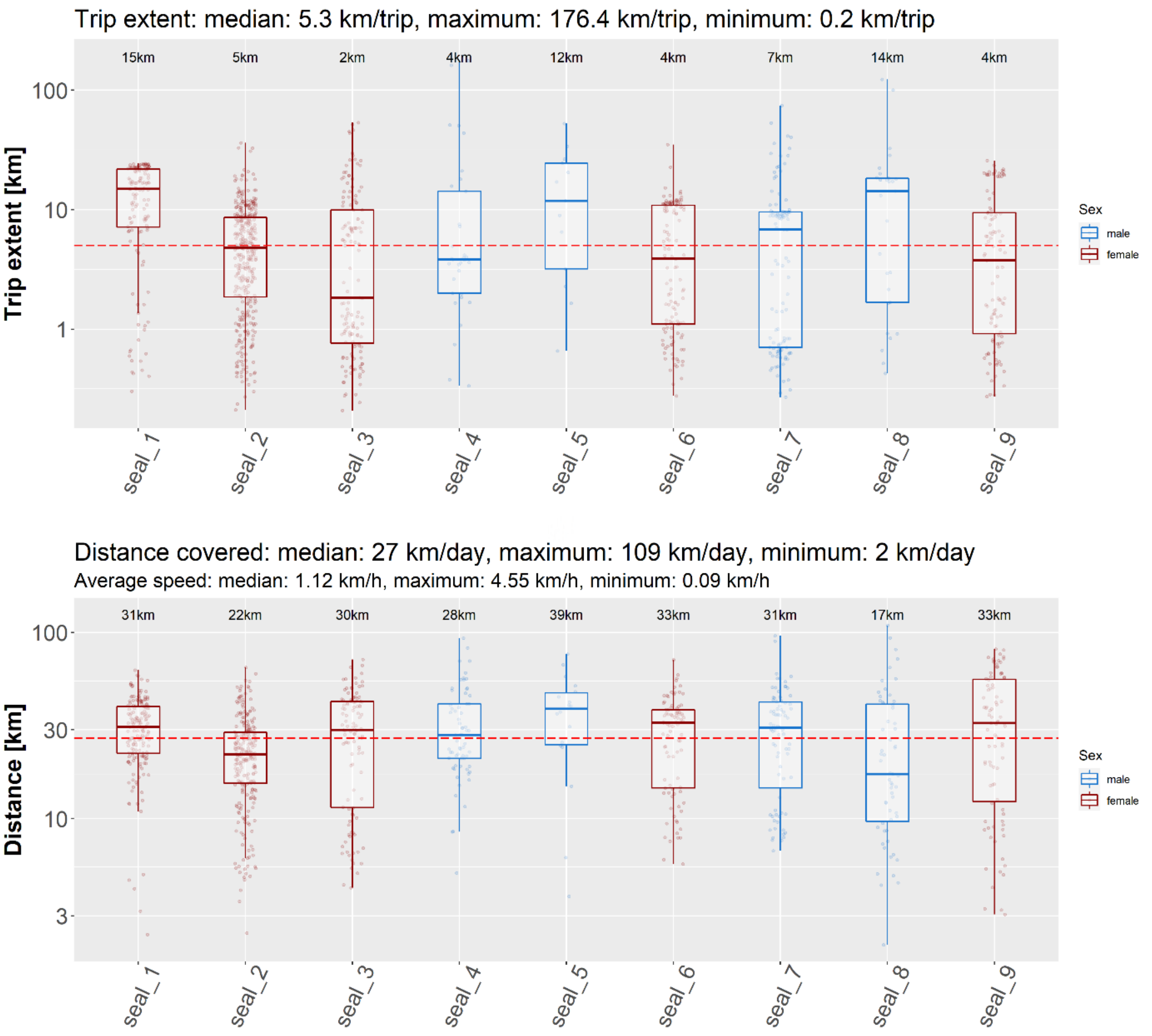

Figure A. 2: Distance covered per day (top) and median trip extent (bottom) per individual colour coded by sex. The data values shown on the y-axis were log transformed to account for outliers present in the data. Boxes represent the 25th and 75th percentiles, the bar the median and whiskers extend from the hinge to the largest or smallest value but no further than 1.5 * the inter-quartile range. The x-axis shows the seal id.

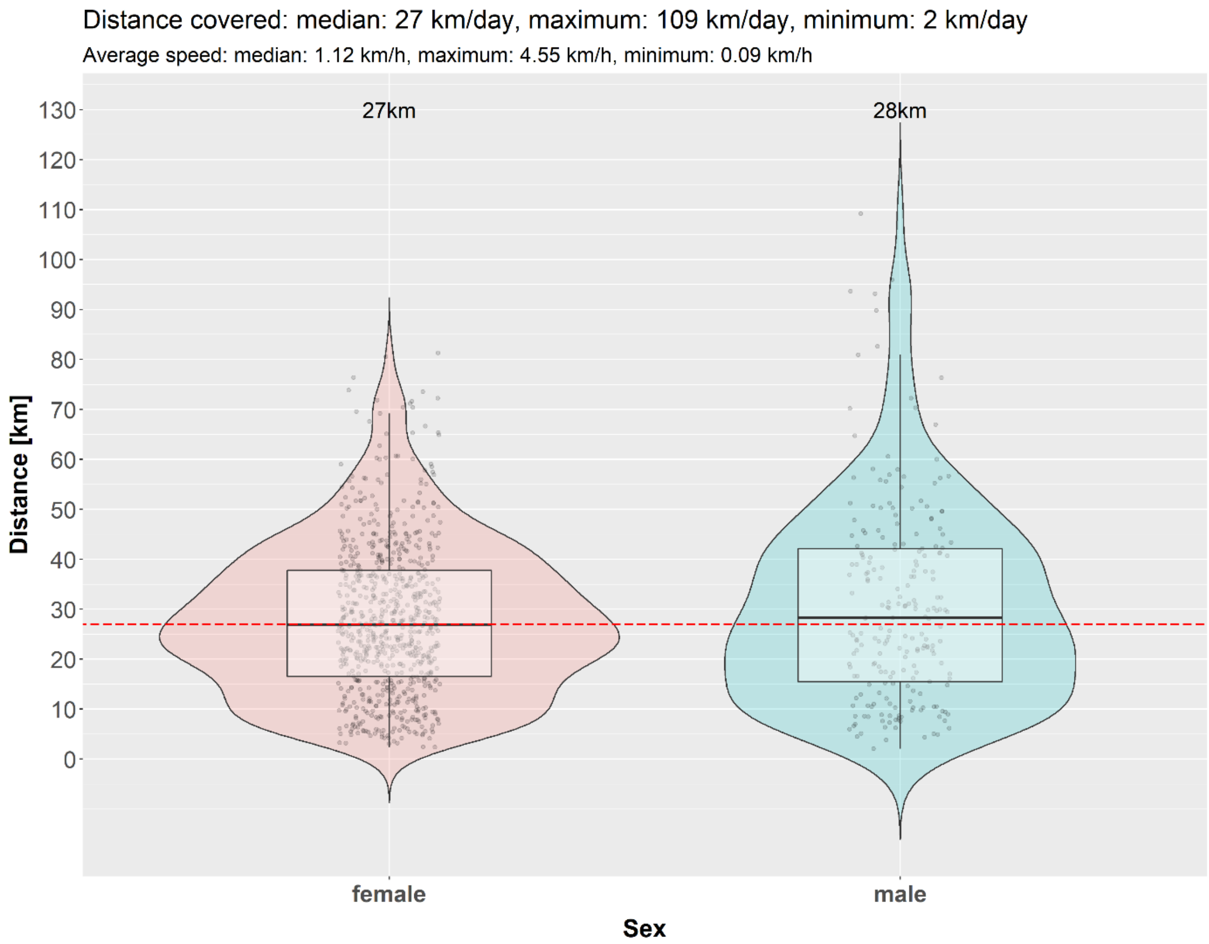

Figure A. 3: Box plot of distances covered per day per individual separated by sex. The violin plot shows the mirrored density distribution of the covered distances. Shown speed values are based on a calculation and therefore not necessarily represent true speed.

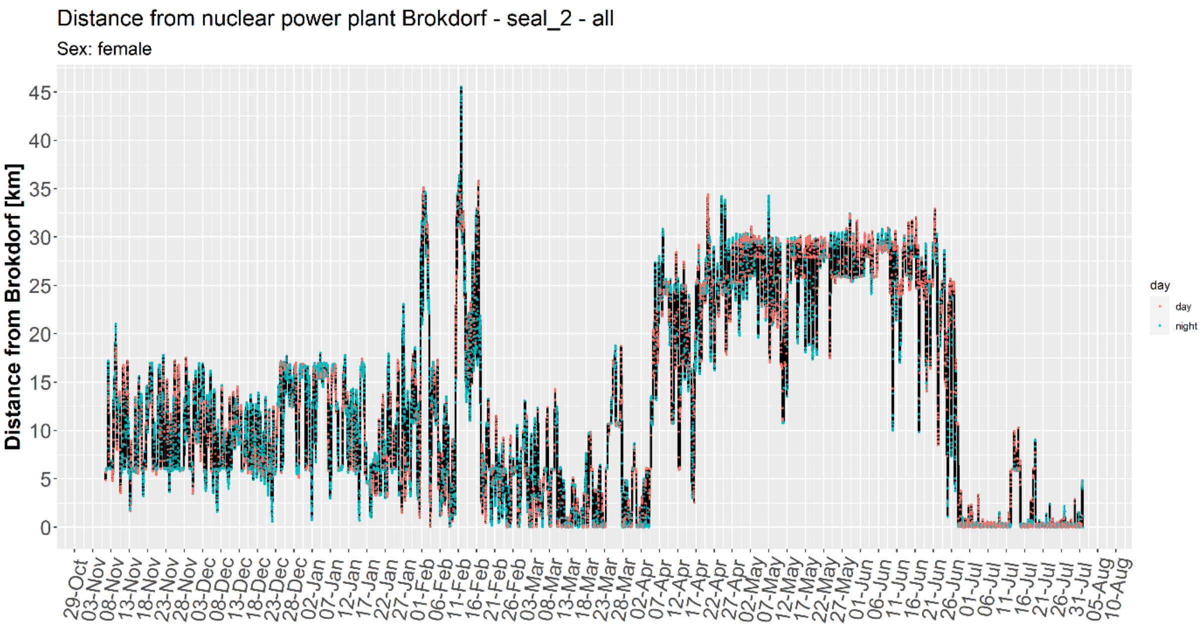

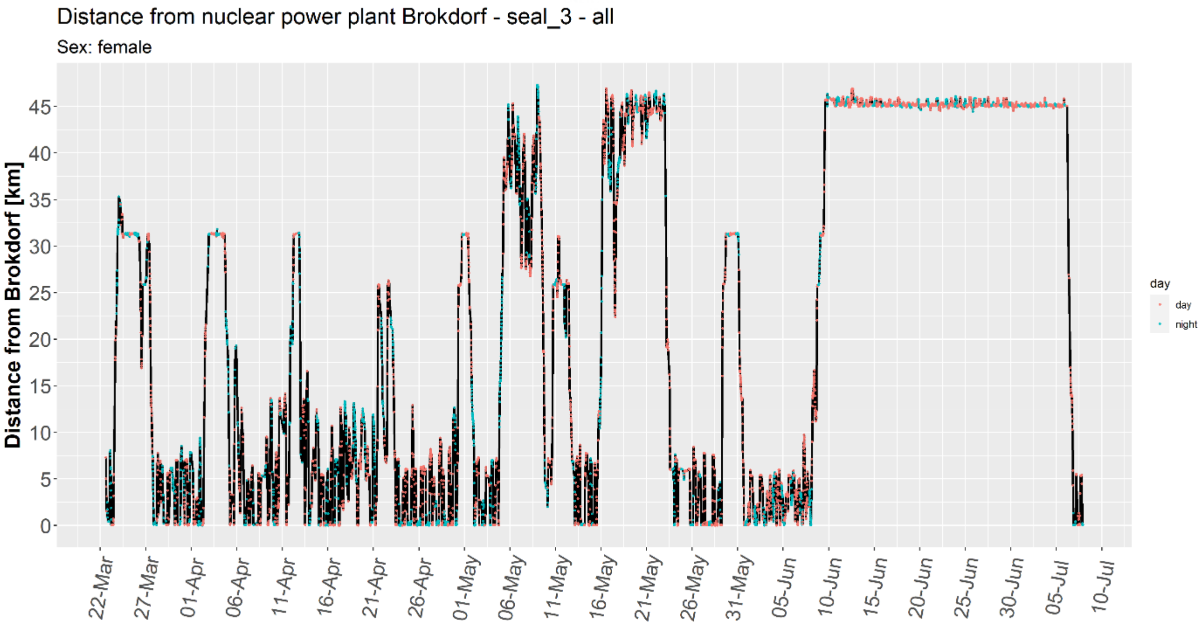

Figure A. 4: Plot showing the distance between the position of the individuals seal_2 (<u>top</u>) and seal_3 (<u>bottom</u>) and the cooling water outlet of the NPP "Brokdorf". Data points are coloured by "day" (between sunrise and sunset) in red and "night" (between sunset and sunrise) in blue.

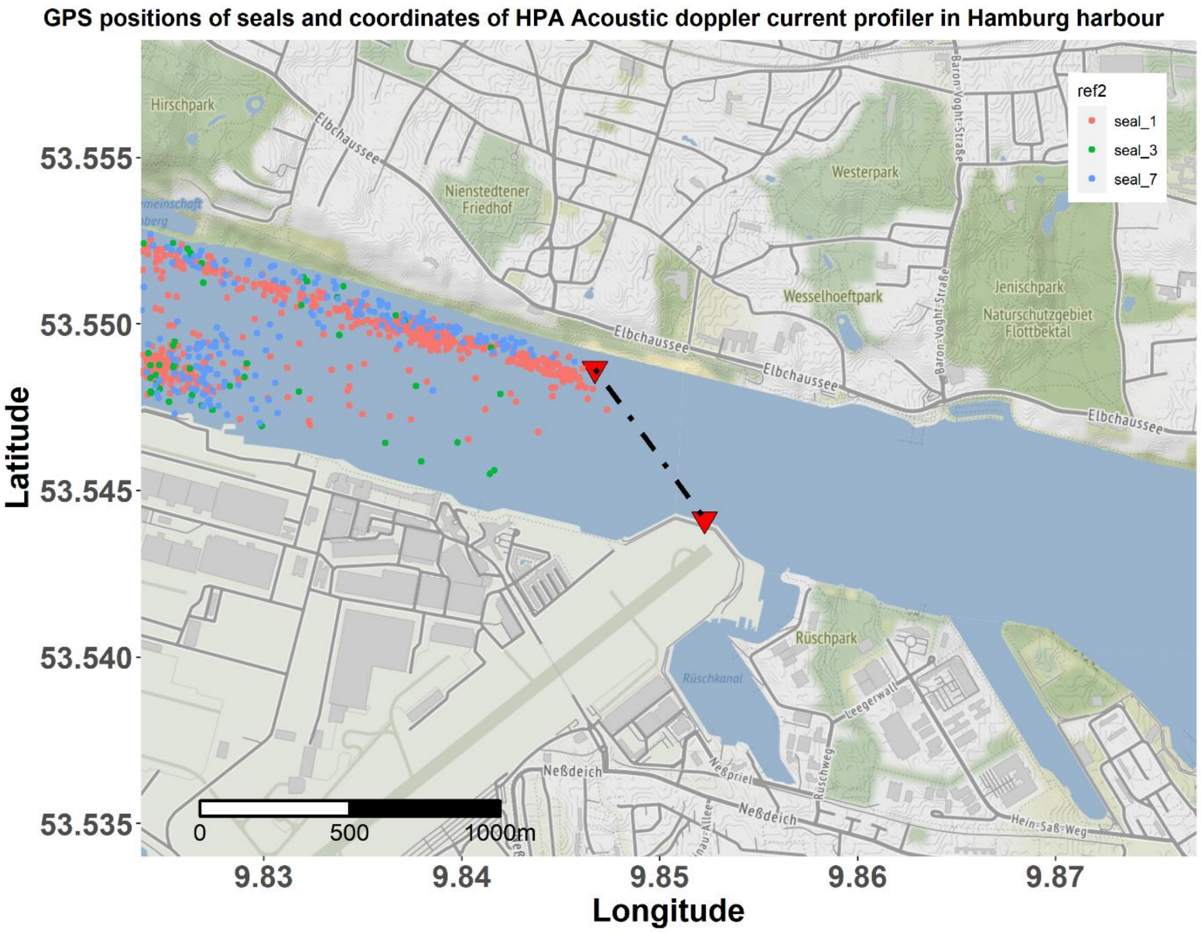

Figure A. 5: Map of the Elbe around river kilometre 631 near Finkenwerder showing recorded GPS positions with the southern limit of the movement of the seals. The red triangles indicate the positions of the acoustic doppler current profiler operating across the river. This map was created using ggmap [63].

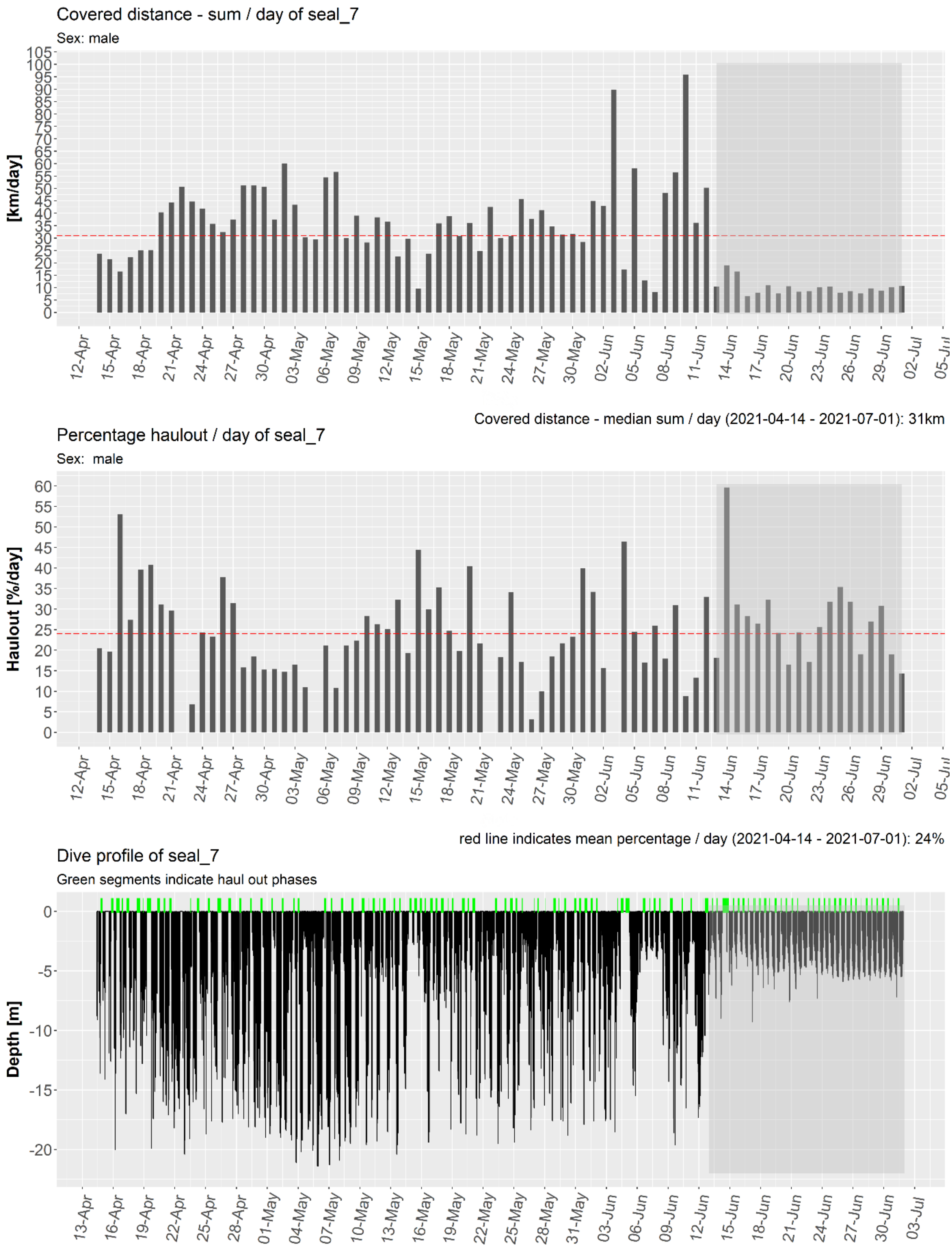

Figure A. 6: Bar plots showing the covered distance per day (<u>top</u>), the proportion of the day spent in haul out (<u>middle</u>) and the dive depth reached with periods of haul out indicated in green (<u>bottom</u>) for the male seal_7 as example. Grey shaded areas indicate the period of behavioural change indicative for the reproductive period.

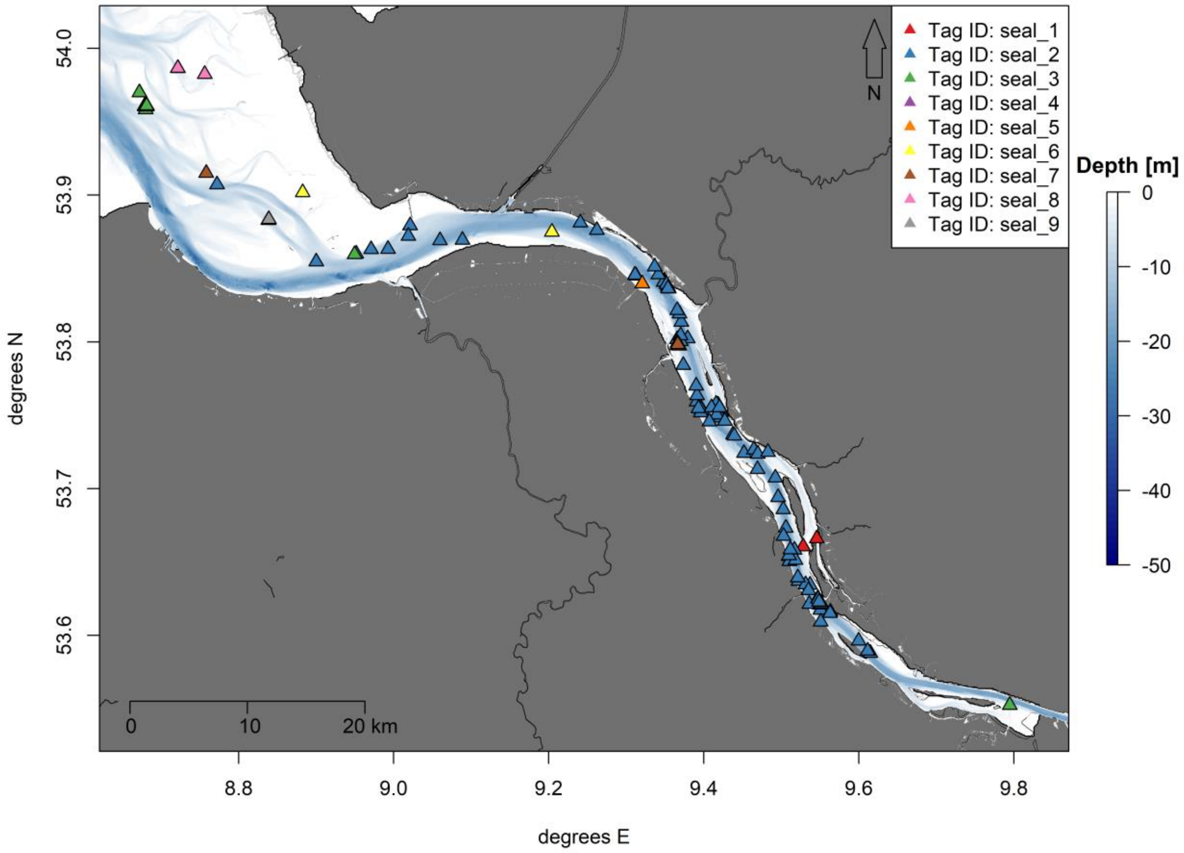

Figure A. 7: Map showing the excluded haul out positions based on a 3.5 m bathymetric threshold.
